# Characterization of Biodegradable Films Made from Taro Peel (*Colocasia esculenta*) Starch

**DOI:** 10.3390/polym15020338

**Published:** 2023-01-09

**Authors:** Rusta Bidari, Annur Ahadi Abdillah, Rogelio Alfredo Bonilla Ponce, Albert Linton Charles

**Affiliations:** 1 International Master’s Degree Program in Food Science, National Pingtung University of Science and Technology, 1 Shuefu Road, Neipu, Pingtung 91201, Taiwan; 2 Department of Tropical Agriculture and International Cooperation, National Pingtung University of Science and Technology, 1 Shuefu Road, Neipu, Pingtung 91201, Taiwan; 3 Department of Marine, Faculty of Fisheries and Marine, Universitas Airlangga, Surabaya 60115, Indonesia

**Keywords:** biodegradable film, chemical pollution, environmental pollution, petrochemical, zero waste

## Abstract

Studies of renewable polymers have highlighted starch’s role to replace petroleum-based components to produce biodegradable films with plastic-like qualities. In this study, the novelty of taro peel starch (TPS) to produce such films using the casting technique is reported for the first time. A response surface method (RSM) approach was employed to optimize different concentrations of TPS (2.5–3.5%, *w*/*w*) and glycerol (25–35%, *w*/*w*) and investigate their effects on the physico-mechanical and water barrier properties of TPS films. TPS films showed a positive linear effect (*p* < 0.05) for thickness (0.058–0.088 mm), opacity (1.95–2.67), water vapor permeability (0.06–0.09 g∙m/m^2^∙kPa∙h), and cubic effect (*p* < 0.05) for moisture content (0.58–1.57%), which were linked to high starch concentrations when plasticized with glycerol. X-ray diffraction analysis of TPS films depicted “amorphous”-type crystalline structure peaks at 19.88°, while the thermogravimetric analysis of the film samples exhibited 75–80% of the weight loss of TPS film in the second phase between temperatures of 300 °C to 400 °C. All films exhibited homogenous, transparent surfaces with flexibility, and completely degraded in 5 days in simulated river water and composting soil environments, which confirmed TPS as a promising film polymer in food packaging.

## 1. Introduction

Disposable plastic packaging materials and other non-biodegradable wastes made from fossil-fuel based chemicals are constantly dumped in landfills and eventually enter niche areas of the environment as microplastics or environmental pollutants. Annually, 300 million tons of plastics are produced and disposed of with only 10% to 13% recycled [1]. Plastic use is still in high demand because of its durability, thermal properties, mechanical properties, and excellent processing ability. However, this demand has created high volumes of waste, and managing its disposal and/or recycling has become a critical issue. This in turn has increased awareness for finding renewable materials that can develop plastic-type films with similar physico-mechanical properties, yet biodegradable, which helps reduce waste, petroleum usage, as well as carbon dioxide emissions. Hence, this study was conducted with the purpose of exploring and investigating cheap and eco-friendly raw materials to substitute conventional petroleum-based components to produce biofilms.

Biodegradable films are manufactured from natural compounds, such as lipids, proteins, fibres, and polysaccharides [2,3,4]. Starch is regarded as one of the strongest raw material candidates for manufacturing “bioplastics” because of its high capacity to form good films, in addition to its low cost, abundance, eco-friendly, and renewable features [5]. Starch-based films have been developed from food materials such as potatoes, rice, corn, taro, and other root tuber starches, and approximately 50% of commercially-used biodegradable plastics are manufactured from starch. The production of starch-based bioplastics is simple, and they are extensively used in packaging applications and in many target industries such as food, pharmaceuticals, textiles, and paper. 

Starch is composed of amylose (linear polysaccharide) and amylopectin (branched polysaccharide) structures and is characterized by high gelatinization temperatures [6]. The amylose and amylopectin polysaccharides present in starch mainly contribute to mechanical and optical properties of starch-based films [7]. The linear construction of amylose poly (α-1,4-glucopyranosyl) in starch generally produces biodegradable plastics with stronger and extremely flexible mechanical properties, while the structure of branched amylopectin poly (α-1,4-glucopyranosyl) produces biodegradable plastics that shows decreased resistance to elongation and tensile strength properties [8].

Taro is an important tuber crop that is considered an excellent source of carbohydrates due to its high starch content [9]. The starch concentration present in the taro tuber on a dry basis is about 70–80%, and the particle size of taro starch is the smallest compared to other root and tuber crop starches [10]. Moreover, the amylopectin component of taro starch is short and retains long chain lengths that help in forming firm gels with high elasticity [11]. Taro starch depicts an A-type crystallinity where the crystalline matrix is tightly packed by double helices, which enables the starch to resist higher heat and shear conditions [8]. Reports of taro starch used to develop bioplastics indicate that taro starch films mixed with increasing increments of bentonite or chitosan demonstrated increased mechanical strength [8,12]. However, since no reports were found in the literature, this study is the first to report the use and investigation of taro peel starch in the formation of biodegradable films, which highlights the novelty of this study. 

The scarcity of reports on the novel use of taro peel starch (TPS) in biodegradable film production motivated this study to develop biofilms from taro peel starch and glycerol combinations and to characterize the biofilms based on their physiochemical, thermal, mechanical, surface morphology, crystallinity, and biodegradable properties.

## 2. Materials and Methods

### 2.1. Raw Materials

Fresh taro peel (*Colocasia esculenta*) was provided by local farmers from Wandan Township, Pingtung County, Taiwan. Food-grade glycerol (Gly) was supplied by Nihon Shiyaku reagent Ltd., Japan. All samples were stored in an electronic drying cabinet (Relative humidity: ±50%; temperature: ±25 °C), and all other chemical reagents used in this study were of analytical grade.

### 2.2. Extraction of Taro Peel Starch

Taro (*Colocasia esculenta*) peel (8 kg of the peel collected) was cleaned, cut, and ground into a paste using a domestic blender (Eupa TSI-9168, Tokyo, Japan). The blended sample was mixed with distilled water (1:2 *w*/*v*) and strained several times with a cheesecloth. The liquid was left to sediment overnight and the supernatant was removed to extract the sedimented starch. The starch was collected, washed, and dried in a hot air oven (Model: DOS-45, Deng Yng-Taiwan) at 30 °C for 48 h. Afterwards, the starch sample was ground using a commercial blender (Yu Chi Machinery Co., Ltd., Chang Hua, Taiwan) into powder and sieved through universal certified sieves (US Standard Sieve Series, ASTME No. 20 and Tyler Standard Sieve Series-20 mesh) in a 0.804-µm then 0.104-µm sieve, respectively, to ensure uniform sized taro peel starch particles. The final sample obtained was packed in re-sealable plastic bags and stored in a digital humidity controller (relative humidity 50% and temperature ±25 °C) for further analysis.

### 2.3. Biodegradable Film Production 

The biodegradable film was prepared following casting techniques from the combination of taro peel starch and glycerol (plasticizer) after the procedures of Abdillah and Charles [13]. Film-forming aliquots were prepared by dissolving starch in distilled water (Milli-Q water purification system, Billerica, MA, USA), which was heated on a hot plate magnetic stirrer with constant stirring (200 rpm) at 130 °C for 12–15 min. After gelatinization of starch, glycerol was added into the film-forming solution with continuous stirring for 3 min. Air bubbles in the resin were removed, and then the solutions (20 mL) were cast into a plastic petri dish (90 mm diameter). The films were dried for 4 h in a hot air oven at 60 °C and then were stored in a desiccator in a digitally-controlled humidity drying cabinet (±25 °C, 50% RH).

The effects of different concentrations of starch (2.5–3.5%) and glycerol (25–35%) on the film properties were evaluated by using response surface method (RSM), with 13 experimental runs, as described in Table 1. The response variables (dependent variables) were thickness, water vapor permeability, water solubility, color and opacity, moisture, tensile strength, elongation at break point, and water contact angle. Due to the novelty of taro peel starch to produce biofilms, RSM was employed in this study to screen and investigate all the possible ingredient formulations to produce films with standardized or expected physico-mechanical properties. The use and recommendations obtained from RSM could potentially reduce production costs by optimizing material volume and combinations and accelerating the process of finding ideal film-forming ratios for future industrial application of this study. Furthermore, RSM was used by Nogueira et al. [5] and Delavari and Ocampo [14] in the production of films with satisfactory results.

### 2.4. Characterization of Taro Peel Starch Films

#### 2.4.1. Visual Aspects

Visual aspect was determined to select films with characteristics such as transparency, homogeneity, smoothness, and flexibility of handling. The film samples were photographed using Samsung S9+, shutter speed (1/60), focus 2.4, and lenses 4.30 mm.

#### 2.4.2. Film Thickness

Ten different regions were measured for film thickness using a digital micrometer (Syntek, Guangdong, China) with an accuracy of ±0.001 mm.

#### 2.4.3. Moisture Content and Solubility in Water

The water solubility of film samples was determined according to the method proposed by Nogueira et al. [5], with modifications. Film samples of 20 mm (in diameter) were cut, weighed, and dried in a hot air oven at 105 °C for 24 h. The dried samples were stored in a desiccator to stabilize for 15 min and weighed. The dehydrated samples were immersed individually into 50-mL beakers filled with distilled water and maintained under slow agitation (75 rpm) for 24 h at 25 ± 2 °C in an isothermal reciprocal water bath shaker (SB 302, Double Eagle Enterprise Ltd., Taiwan). Then, insoluble samples were removed and dried at 105 °C for 24 h to determine the final dry mass. Moisture content and solubility in water were calculated according to Equations (1) and (2), respectively:

(1) $$Moisture\;content\;\left(\%\right)=\left[\left(Wi-Wf\right)\times 100\right]$$

(2) $$Water\;Solubility\;\left(\%\right)=\left[\left(\frac{Wi-Ws}{Wi}\right)\times 100\right]$$

where: ‘*Wi*’ is the initial mass of films (g), ‘*Wf*’ is the weight of dry film (g) after first dry, and ‘*Ws*’ is the weight of final insoluble dry films (g).

#### 2.4.4. Opacity

Opacity of the film samples were determined following the procedures of Abdillah et al. [15] and Homthawornchoo et al. [16], by using DU 730 UV/Vis spectrophotometer (Beckman-Coulter, Brea, CA, USA) with slight modifications. The opacity values were calculated using Equation (3):

(3) $$Opacity=\left[\frac{A_{600}}{Thickness\;\left(mm\right)}\right]$$

where: *A*_600_ is the absorbance of taro starch films at 600 nm.

#### 2.4.5. Water Vapor Permeability (WVP) 

The water vapor permeability (WVP) was determined according to the method proposed by Abdillah and Charles [13] and Charles et al. [17]. Films of known thickness were tied with rubber bands over the mouth of glass cups (depth = 70 mm, diameter = 30 mm) containing 10 g of dry silica gels. The glass cups were weighed and placed inside the desiccator with 100 mL of distilled water and a humidity and temperature sensor. The air inside the desiccator was removed and placed inside a digital humidity controller (RH = 50%, temperature = 25 °C). The weight of glass cups was measured, and relative humidity and temperature was recorded at 24-h intervals for 7 days. Water vapor permeability values are expressed in g∙mm/m^2^∙kPa∙h and were calculated using Equations (4) and (5), respectively:

(4) $$\mathrm{WVTR}\;\left(g\;/m^{2}\cdot h\right)=\left[\frac{slope}{film\;area}\right]$$

(5) $$\mathrm{WVP}\;\left(g\cdot \mathrm{mm}/m^{2}\cdot h\cdot \mathrm{kPa}\right)=\left[\frac{WVTR\times thickness\;\left(mm\right)}{WS\times \left(R2-R1\right)}\right]$$

where: *WVTR* is the water vapor transmission rate (g/m^2^∙h); *WS* is water saturation pressure (kPa) at a test chamber; *R*1 is relative humidity of dried silica; and *R*2 is relative humidity of the test chamber.

#### 2.4.6. Tensile Strength and Elongation at Break

Tensile strength (TS) and elongation at break (EAB) were determined using a universal testing machine (YD-TA Jing Koou Enterprice, Kaohsiung, Taiwan) based on the method by the American Standard Testing and Material (ASTM) D-882-02 method [18]. The film samples of known thickness were cut into strips (25 × 7 mm) and fixed by two distal grips with 50 mm grip separation. The TS and EAB of film samples were measured at a crosshead speed of 5 mm/s.

### 2.5. Microstructure of TPS Film

Surface morphology and cross-section of TPS film were observed using SEM-EDX (Hitachi S-300N, Tokyo, Japan) at 500× and 1000× magnification. Small pieces of films were dusted onto a double-sided adhesive tape fixed on an aluminum plate. The samples were coated with a thin layer of gold and subjected to an electronic beam accelerating at a voltage of 10 kV to measure film morphology.

### 2.6. X-ray Diffraction (XRD) of TPS Film

X-ray diffraction (XRD) profile of taro peel starch film was obtained using an X-ray diffractometer (Bruker D8 Advance, Germany) analyzer following the method by [13], with Cu Kα at 40 kV and 40 mA. The film sample was scanned from 5 = 40 °C with a scan rate of 10° min^−1^, and XRD patterns of taro peel starch film are expressed as peak intensity (a.u) from different 2θ (°).

### 2.7. Thermogravimetric Analysis (TGA) of TPS Film

Thermogravimetric analysis of taro peel starch film sample was characterized using thermogravimetric analyzer (Perkin Elmer Taiwan Cooperation, Taipei, Taiwan) following the method by [17]. Each sample (~7 to 8 mg) was weighed in an aluminum sample pan and heated under nitrogen atmosphere from 25–600 °C with a heating rate of 10 °C/min to measure the degradation of starch.

### 2.8. Fourier Transform Infrared Analysis (FTIR) of TPS Film 

Fourier transform infrared analysis (FTIR) of taro peel starch film sample was obtained using FTIR spectrophotometer (Perkin Elmer-Spectrum 100 FT-IR, Waltham, MA, USA). The FTIR sample analyzed was expressed as transmittance (%) in a range of wavelength between 500 and 4000 cm^−1^ based on the method by [15]. 

### 2.9. Biodegradability Test

River water collected from Wanan Riverside Park, Taiwu Township, Pingtung County, Taiwan, was used to evaluate the biodegradability of TPS film samples in river water conditions. TPS film samples strips (20 mm × 20 mm) were immersed in 30 mL of river water and evaluated every day until its complete degradation.

In another experiment, soil biodegradability of TPS film samples was qualitatively conducted in composting soil (purchased from a local plant and flower market, Neipu, Pingtung, Taiwan). TPS film samples (20 mm × 20 mm) were buried (20 mm) in composting soil and kept outdoors to evaluate its complete degradation based on the method by [19] with some modifications. The moisture of the composite was maintained by spraying water once a day and evaluation was performed every 5 days and documented using Samsung S22 Ultra, shutter speed (1/24), focus 2.2, and lenses 2.20 mm.

### 2.10. Statistical Analysis

Experimental data were statistically analyzed using the Central Composite Design (Design Expert Software version 13, StatEase Inc., Minneapolis, MN, USA). Numerous statistical parameters (lack-of-fit, predicted and adjusted multiple correlation coefficients, and coefficients of variation) of different polynomial (linear) models were compared to select the best fitting polynomial (linear) model. Significant difference was determined through analysis of variance (ANOVA) by calculating F-value at *p* < 0.05 significance level.

## 3. Results and Discussions

### 3.1. Characterization of Taro Peel Starch Films

#### 3.1.1. Visual Aspects

**Figure 1:**
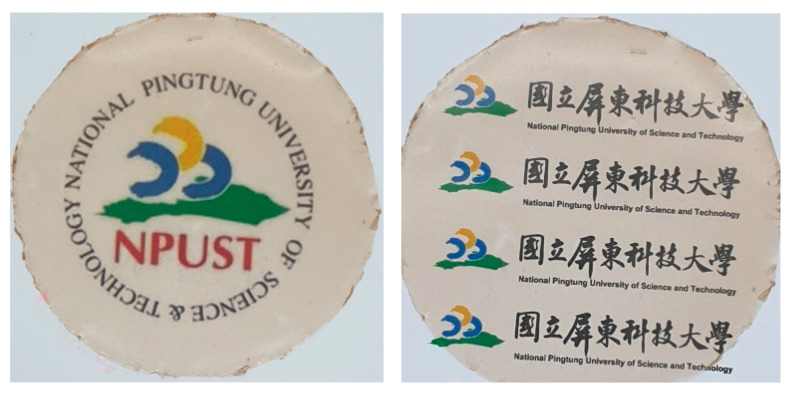
Visual appearance of taro peel starch film using different logos.

All taro peel starch film samples presented homogeneous surfaces without bubbles and insoluble particles (Figure 1). The film samples were odorless, transparent, and brown in color. Moreover, the samples demonstrated flexibility and could be easily removed from the petri-dishes. Addition of glycerol in the film creates high tendency to absorb the water molecules due to its three hydroxyl groups, and the internal hydrogen bonding of the film was reduced by glycerol, which led to increased inter-molecular space; as a result, the film became transparent and flexible [20].

#### 3.1.2. Film Thickness

The thickness of TPS films was recorded between 0.058 mm to 0.088 mm. Among the film samples investigated based on ratios of TPS and glycerol concentrations, the maximum thickness (0.088 mm) was observed for the combinations of 3.70% starch and 30% glycerol concentrations, respectively, followed by 3.5% starch with 35% glycerol concentration, respectively, whereas minimum thickness (0.058 mm) was observed for the combination of 2.29% starch and 30% glycerol concentrations, respectively.

**Figure 2:**
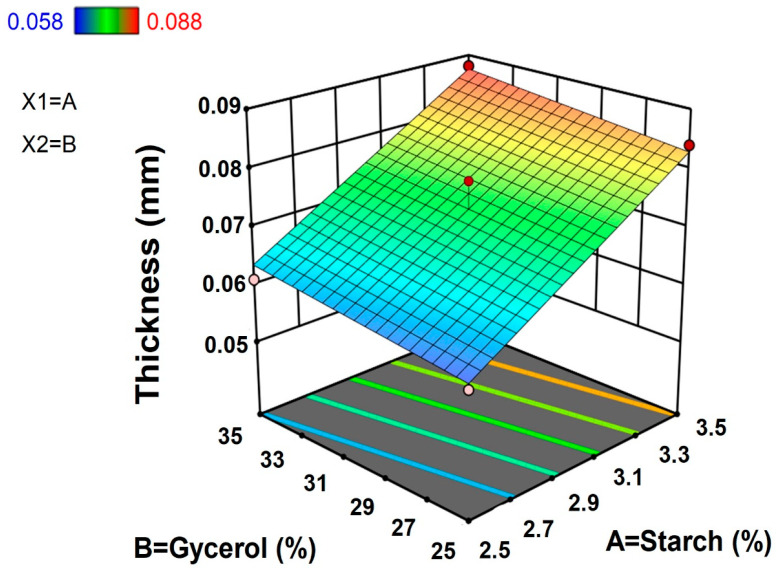
Response surface plot for thickness of taro peel starch biodegradable films.

The results from Table 2 and Figure 2 indicate that concentrations of TPS and glycerol had positive linear effects (*p* < 0.05) on films’ thickness, that is, increases in concentrations resulted in increased film thickness. It was also observed that film thickness positively correlated more with TPS than glycerol. Film thickness of either arrowroot starch [5] and/or potato peel starch [17] was also influenced by increased starch concentrations in films, which resulted in increased thickness when plasticized with glycerol. In this study, concentrations of starch and glycerol highly influenced film thickness, which then affected the films’ mechanical characteristics such as tensile strength, elongation, and barrier properties. The increase in thickness might be due to large amount of mass of starch with a larger surface area, which improves when interacted with the plasticizer [21]. In addition, the role of the plasticizer is attributed to disrupting and restructuring of the inter-molecular polymer chain network, which creates more free volume resulting in a thicker film. Thickness is an important aspect of films as it is related to its barrier properties. Since starch-based films are hydrophilic, film thickness might influence water vapor permeability due to differences between moisture accumulation and water vapor pressure at the film interface [22]. 

#### 3.1.3. Moisture Content and Solubility in Water

Moisture content plays a crucial role in the use of films for food packaging since moisture content influences the shelf life and texture of foods [17]. The moisture content of taro peel starch (TPS) films ranged from 0.58% to 1.57%. The maximum moisture content (1.57%) was observed for the combination of 3% starch concentration and 37.07% glycerol concentration, whereas the minimum moisture content (0.58%) was observed for the combination of 3% starch concentration and 22.93% glycerol concentration. Taro peel starch-based film showed lower moisture content compared to the biodegradable film from potato peel starch (11.53% to 12.98%) reported by Charles et al. [17].

**Figure 3:**
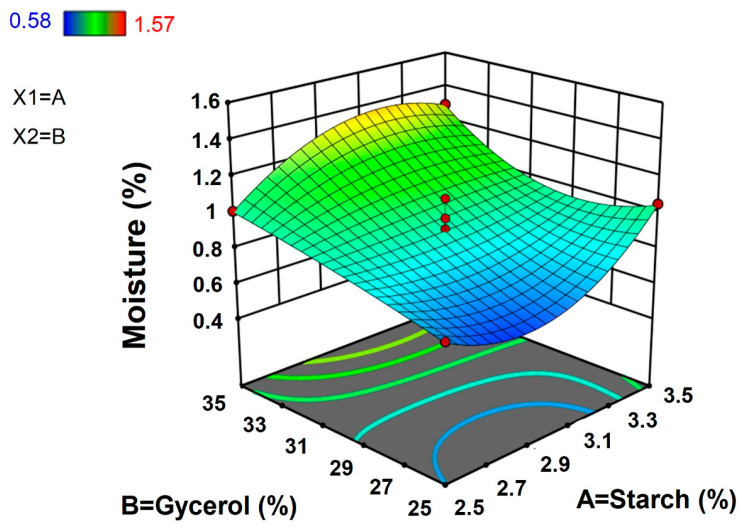
Response surface plot for moisture content of taro peel starch biodegradable films.

The results from Figure 3 showed that moisture content of the films increased with higher concentrations of TPS and glycerol, respectively. The results of equation of response, regression analysis, and ANOVA for moisture content of taro peel starch are presented in Table 2 and Table 3 and suggested that starch and glycerol concentrations had significantly positive cubic effects (*p* < 0.05) on moisture content of films.

Meanwhile, the water solubility of taro starch film ranged between 3.6% and 19.13%. The maximum water solubility (19.13%) was observed for the combination of 3.5% starch combination and 25% glycerol concentration, and minimum water solubility (3.6%) was observed for the combination of 2.5% starch concentration and 35% glycerol concentration. However, analysis of variance showed that starch and glycerol had a non-significant effect (*p* > 0.05) on films’ solubility (Table S1).

#### 3.1.4. Opacity

Color and opacity of the biodegradable film are important measures for acceptability as packaging materials. Normally, for good visual presentation of products, films intended for packaging should have high gloss and low opacity [23]. The opacity of TPS films was recorded between 1.95 and 2.67, and among the film samples investigated based on ratios of TPS and glycerol concentrations, the maximum opacity (2.67) was observed for the combination of 2.5% starch concentration and 25% glycerol concentration, whereas minimum opacity (1.95) was observed for the combination of 3.5% starch concentration and 25% glycerol concentration.

**Figure 4:**
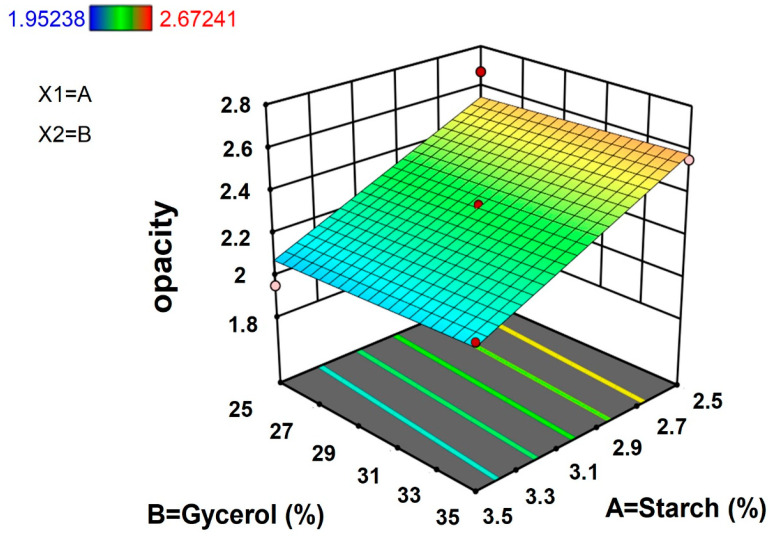
Response surface plot for opacity of taro peel starch biodegradable films.

The results from Figure 4 showed that lower concentrations of TPS and higher concentration of glycerol increased the films’ opacity. However, when the concentration of starch increased, the films’ opacity decreased. The reduction of opacity was attributed to cross-linking of the starch molecules, which can decrease the crystallinity of starch with an eventual decrease in opacity [24]. The results of the equation of the response, regression analysis, and ANOVA for the opacity of TPS are summarized in Table 2 and Table 3. The results demonstrated that the starch and glycerol concentration had positive linear effects (R^2^ = 0.8482) on the opacity of films.

#### 3.1.5. Water Vapor Permeability 

Water vapor permeability (WVP) plays an important role in the characterization of functional properties of polysaccharide-based biofilms such as their biodegradability. WVP is the ability of water molecules to penetrate the material matrix and is used to evaluate the capacity of the material to block water [25]. Hence, the WVP of the films should be lower to extend the shelf-life of the packaged food. The WVP of TPS films ranged from 0.056 to 0.091 g∙mm/m^2^∙kPa∙h. Maximum WVP (0.091 g∙mm/m^2^∙kPa∙h) was observed for the combination of 3.5% starch concentration and 35% of glycerol concentration whereas a minimum WVP (0.056 g∙mm/m^2^∙kPa∙h) was observed for the combination of 2.29% TPS concentration and 30% glycerol. The increase in the amount of dry matter (starch) increased the water vapor permeability of TPS films, which was supported by results of the water contact angle test (Figure S1). 

**Figure 5:**
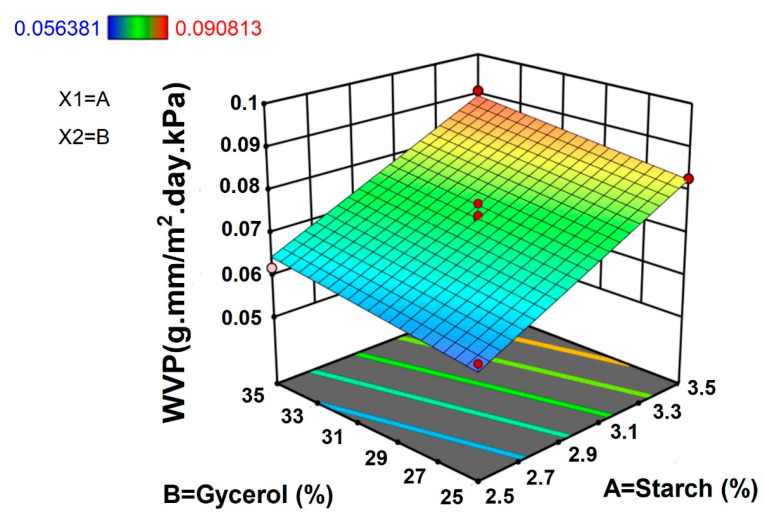
Response surface plot for water vapor permeability of taro peel starch biodegradable films.

The results from Figure 5 showed that higher concentrations of TPS and a higher concentration of glycerol increased the WVP of films. The equation of response, regression analysis, and ANOVA results WVP of taro peel starch films are presented in Table 2 and Table 3. The results confirmed that starch and glycerol concentration had a positive linear impact (R^2^ = 0.973) on WVP of the films. According to Cazón et al. [26], starch-based films have exceptional barrier properties because of their highly-ordered hydrogen-bonded network structure in which the amylose and amylopectin form crystalline and non-crystalline regions in alternating layers. In addition, Gutiérrez et al. [22] reported that thicker films increased the resistance to water transfer and partial pressure along the internal surface of the films at equilibrium. Plasticizers (such as glycerol) consisting of a higher number of hydroxyl groups increase the free space among the polymers with increasing polymer mobility and cause films to have hydrophilic property, which eventually affect the increase of water vapor transmission rate of the films [27]. Therefore, factors such as source and proportion of hydrocolloids, plasticizers, and differences in thickness could affect the WVP of films.

#### 3.1.6. Tensile Strength and Elongation at Break

The mechanical properties of films are important parameters to evaluate if the films can be used as food packaging materials [28]. The tensile strength of taro peel starch ranged from 0.66 MPa and 1.63 MPa. The maximum tensile strength (1.63 MPa) was observed for the combination of 2.5% starch concentration and 35% glycerol concentration whereas a minimum tensile strength (0.66 MPa) was observed for the combination of 3% starch concentration and 30% glycerol concentration. Meanwhile, the elongation at break linearly decreased with increases of TPS in the films, which ranged from 78.8% to 263.6%. The maximum elongation at break (263.6%) was observed for the combination of 2.5% starch concentration and 35% glycerol concentration and minimum elongation at break (78.8%) was observed for the combination of 3% TPS concentration and 22.93% glycerol concentration.

The lower concentrations of TPS and higher concentrations of glycerol showed increases in both tensile strength and elongation at break of TPS films. Analysis of variance showed that TPS and glycerol had non-significant effects (*p* > 0.05) on tensile strength and elongation at break (Table S1). 

In unreported experiments, TPS exhibited an amylose content of 14.15 ± 0.063%, which is reportedly lower among root crop starches [29]. According to Muscat et al. [30], starch-based films show higher tensile strength and lower elongation at break when starch with high amylose content was used to produce the films. However, both low and high amylose content starch films showed decreases in tensile strength and increases in elongation when the plasticizer concentration increased (higher than 15%). Furthermore, the plasticizer reduces the intramolecular attraction force between the starch chains that promotes hydrogen bond formation among glycerol and starch molecules, resulting in a decrease of tensile strength and greater flexibility. Furthermore, starch films with high amylose content usually have a greater crystalline domain compared to that of films with low amylose content, leading to greater mechanical resistance [31]. These domains could act as reinforcement for enhancing the films’ mechanical properties as they are fixed in an amorphous matrix. In addition, Muscat et al. [30], Domene-López et al. [31], and Cano et al. [32], reported that higher molecular weight and amylose content of the starch resulted in higher crystallinity of the films, which eventually increased the tensile strength and decreased the elongation of break of the starch films. Biodegradable films made from starch generally have lower tensile strength than common plastic films, while their elongation-at-break varies widely [33]. Moreover, temperature also plays important roles that influence the physical and mechanical properties of films. According to Han [33], the tensile strength of films below 1 MPa or between 1–10 MPa is regarded as inferior or marginal for physical strength of plastics. Therefore, the proximate resin of starch and other polymers could be used to enhance the physical strength of biodegradable films [8,13].

### 3.2. Microstructure of TPS Film

**Figure 6:**
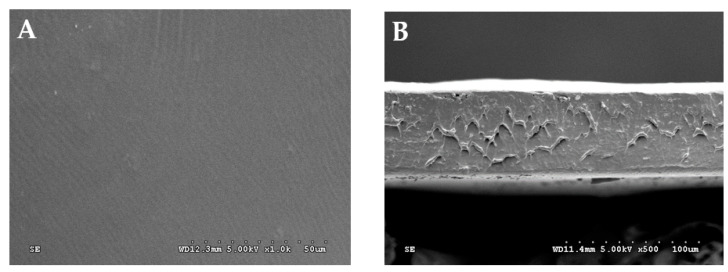
SEM micrograph of surface of taro peel starch, and glycerol blend-based membranes (**A**) film surface at magnification power of 1000×, and (**B**) cross-sectional of films at magnification power of 500×.

SEM micrographs of the optimum film sample (2.95% starch and 31.84% glycerol) exhibited a compact morphology without any pores and cracks. The film sample exhibited a smoother and uniform surface structure as shown in Figure 6. According to Gómez-Aldapa et al. [34], starch with smaller granules increases its contact area when interacting with water molecules and polymeric material, which promotes improved gelatinization of starch. When the gelatinized solution is dried, the films appear more homogenous due to lower number of remaining granules. However, the cross-section (Figure 6B) of the film sample exhibited fractured areas due to poor interaction between glycerol and the starch, which confirmed that the morphological properties of the film might be influenced by the preparation technique of the resin [19]. The heterogenous fractured area might also be due to crystallization in that region, which is linked with water vapor diffusion caused by molecular mobility near the film surface [32]. Therefore, the enhanced network of the film matrix link could help to improve the mechanical properties of the taro peel starch films.

### 3.3. X-ray Diffraction (XRD) of TPS Film

**Figure 7:**
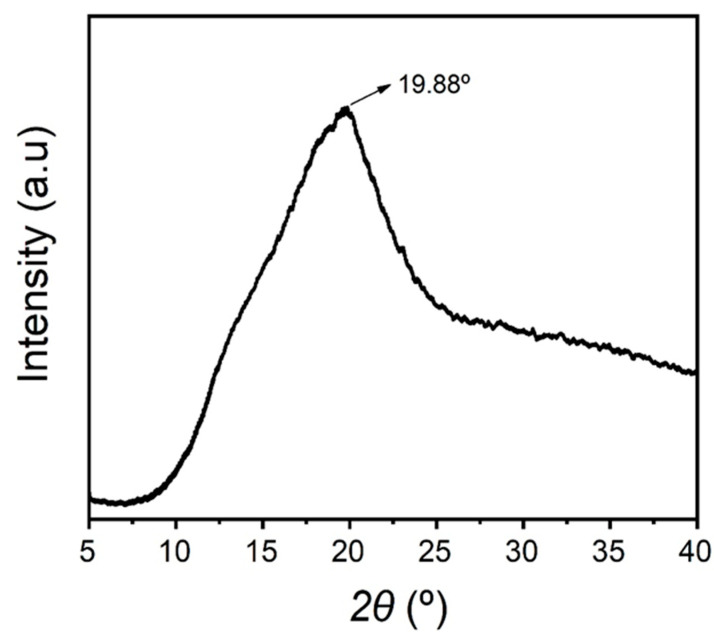
X-ray diffractometry (XRD) of taro peel starch film.

The XRD diffraction pattern (Figure 7) of taro peel starch film (2.95% starch and 31.84% glycerol) indicated enhanced reduction in crystalline intensities that could be characterized as amorphous form. The peak intensity was observed at 19.88°, which represented the amorphous crystalline nature of the film samples. Meanwhile, the results of thermoplastic recycled carbon ashes/maize starch (TPAS) composite films showed the semi-crystalline nature of all starch films, both in the presence and absence of ashes [35]. According to Abdillah and Charles [13] and Charles et al. [17], the amorphous nature of the film might be induced during the process of the casting technique, where the addition of glycerol decreases the intermolecular hydrogen bonding (by inhibiting starch retrogradation), which increased in the chain mobility of starch molecules. Furthermore, during the casting method of the film, the resin (starch and water mixture) was heated until its (starch) gelatinization, where the crystalline structure of starch is completely destroyed. As a result of this irreversible process, changes in the crystalline nature of the films were observed to be amorphous. In addition, the film representing the amorphous form are soft and flexible, which could be considered suitable in food packaging application [17]. 

### 3.4. Thermogravimetric Analysis (TGA) of TPS Film

**Figure 8:**
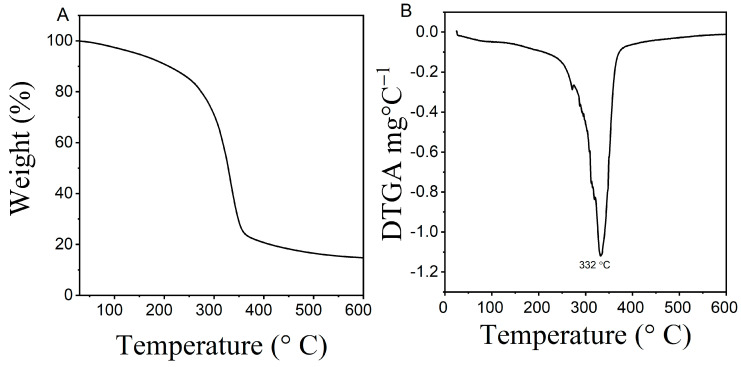
(**A**) TGA, and (**B**) DTGA thermograms for taro peel starch film.

The TGA and DTGA thermograms (Figure 8) illustrates the thermal stability of the taro peel starch film sample (2.95% starch and 31.84% glycerol), where thermal degradation was observed between 50–600 °C. The first phase occurred within the temperature range of 50–300 °C depicted by water evaporation (water weight loss) and vaporization of glycerol, which resulted in 15–20% weight loss of the sample. The second and third phase depicted a decomposition temperature between 300–400 °C. The weight loss was related to saccharide degradation and carbonization of organic matter with complete oxidation until 600 °C [36]. As a result, 75–80% of sample weight was lost and the remaining 20–25% was char and ash residues [17]. The derivate TGA curve indicated the peak curve for taro peel starch at 332 °C. Therefore, based on these results, taro peel starch film was concluded thermally stable and possessed desirable characteristics as biodegradable packaging films.

### 3.5. Fourier Transform Infrared Analysis (FTIR) of TPS Film

**Figure 9:**
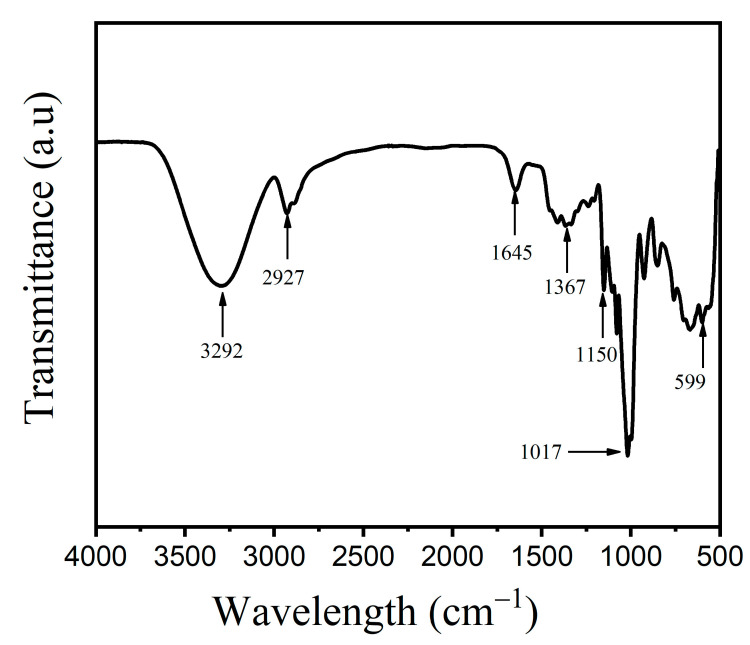
Fourier transform infrared (FTIR) spectra of taro peel starch film.

The FTIR absorption bands of functional groups present in taro peel starch film is illustrated in Figure 9. The absorption peak observed at 3292 cm^−1^ indicated the presence of hydroxyl groups (OH group) and H-bonded OH stretching of the taro peel starch. The absorption peak observed at 2927 cm^−1^ suggested the presence of C-H groups, which were depicted as characteristic of starch, glycerol, and the glucose units present in the sample. Similarly, Sohany et al. [37] observed absorption peaks for sweet potato starch and sweet potato peel films at 3200–3500 cm^−1^ which were ascribed to strong stretching vibration of massive hydroxyl (O-H) groups, and peaks at 2927–2934 cm^−1^ were attributed to stretching vibration of methyl (C-H) groups. According to Shanmathy et al. [8], the FTIR peaks that appeared broadened was attributed to the absorption of water molecules by starch granules. In this study, the absorption band of carbonyl of non-substituent amide and water content was observed at the peak of 1645 cm^−1^ and absorption band of carboxyl group was observed at peak of 1367 cm^−1^. The absorption band observed in the range of 1018–1155 cm^−1^ was related to the peaks of pyranose form of the glucose residue [38] and C-I stretching (Halo group) was observed at peak 1016 cm^−1^. Furthermore, the results of thermoplastic cassava starch films confirmed the presence of absorption peaks located at 1018 cm^−1^, which were assigned to C-O stretching vibration of the C-O-C groups in glucose units [39]. Additionally, according to Moustafa et al. [6], the FTIR spectra of starch typically showed an extremely broad band at 3387 cm^−1^, and the band at 2930 cm^−1^ was attributed to O–H stretching and C–H stretching vibrations, respectively. The presence of hydroxide and carbonyl groups meant that taro peel starch-based film had better biodegradable properties [12]. Similar results were observed for Abdillah and Charles [13], where film ‘A’ made with combination of arrowroot starch and glycerol exhibited similar absorption bands and peaks.

### 3.6. Biodegradability Test

**Figure 10:**
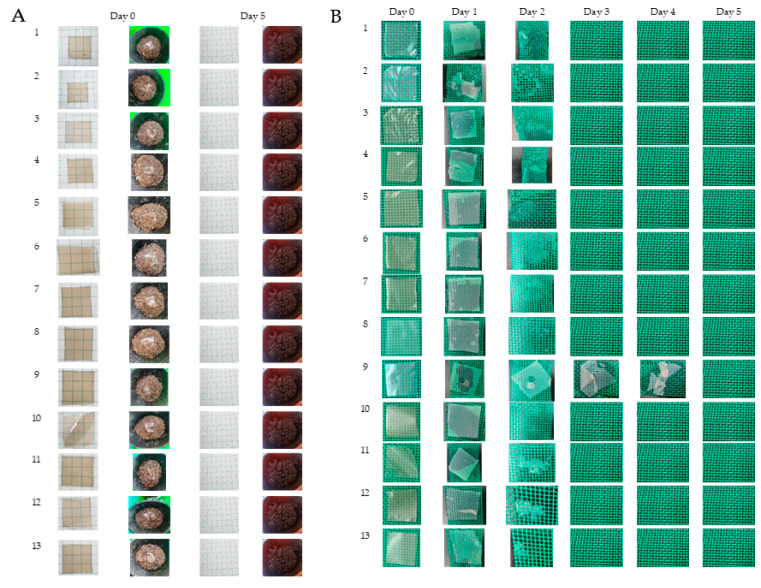
(**A**) Composting soil biodegradability, (**B**) river water biodegradability test of taro peel starch (TPS) films.

Biodegradation is described as loss of physico-mechanical properties, fragmentation of the sample, and chemical modification caused by presence of enzymes and microorganisms in nature [19]. Film biodegradation in soil and river water was conducted to determine the films’ degradation times and confirm the potential of TPS as substitutes for petroleum-based components, which require longer periods for ‘degradation’ in the environment. The qualitative test for composting soil was based on visual assessment where biodegraded films were observed at day 0 and day 5 as illustrated in Figure 10A. The fastest time for compost soil degradation was observed on day 5. Biodegradable films consisting of hydroxyl and carboxyl functional groups as flexible active sites degrade faster since these functional groups enable the films to attach on enzyme sites [40]. Additionally, polymers with shorter chains degrade faster than the polymer with complex chemical structures [41]. According to Abdillah and Charles [13] and Medina-Jaramillo et al. [42], the rich microflora present in the composting soil possibly assisted with accelerated degradation of the films. Furthermore, Charles et al. [17] stated that fungi are accountable for soil biodegradation, while bacteria are dominant in aquatic habitats.

The biodegradation of the films in river water was also completed in 5 days as shown in Figure 10B. All the samples had completely solubilized and degraded by day 3 except sample 9 (3% starch and 37.0711% glycerol), which started fragmentation on day 3 and completed biodegradation by day 5. However, the addition of the plasticizer assisted in easier and faster degradation of the films [43,44]. Nevertheless, all the film samples met the requirement of biodegradation established by (ASTM) D-6002 method [45], which standardizes complete degradation in 60 days. Thus, all film samples were completely degraded, which confirmed the films were biocompatible, perishable, non-toxic, and potentially suitable in the development of films intended for use as food packaging materials.

## 4. Conclusions

Taro peel starch (TPS) film samples exhibited transparent, homogenous, and flexible surfaces without insoluble particles; moreover, TPS films exhibited an amorphous crystalline nature. Thermogravimetric analysis demonstrated that approximately 75–80% of mass loss was attributed to saccharide degradation and carbonization of the organic matter, which occurred between 300 to 400 °C. This study revealed that the films developed from increased increments of TPS plasticized with glycerol exhibited positive linear effects (*p* < 0.05) for thickness (0.058–0.088 mm), opacity (1.95–2.67), water vapor permeability (0.06–0.09 g∙mm/m^2^∙kPa∙h), and cubic effect (*p* < 0.05) for moisture content (0.58–1.57%). However, the limitations of analysis of variance on tensile strength, elongation at break, and water solubility showed that TPS and glycerol had non-significant effects (*p* > 0.05). In addition, all TPS films completed river water and composting soil biodegradation within 5 days. Moreover, a major feature of this study was the transformation and or bioconversion of waste (taro peel) using a low-energy and eco-friendly extraction method to produce starch (taro peel starch), which was further converted into biodegradable films. However, the films produced were not as durable compared to other biodegradable films produced by other plant-based polymers. Nevertheless, it was concluded that taro peel starch could potentially be a low-cost renewable polymer to produce biodegradable film materials. Hence, composite resins with taro peel starch and other renewable polymers (for instance carrageenan) will be investigated to design and develop prototype packaging products with a wider range of application in the food and packaging industries.

## Figures and Tables

**Table 1:** Statistical project for the development of biodegradable film from taro peel starch using RSM software.

	Factor 1	Factor 2	Response 1	Response 2	Response 3	Response 4	Response 5	Response 6	Response 7
Runs	A Starch (%)	B Glycerol (%)	Thickness (mm)	Tensile Strength (MPa)	Elongation at Break (%)	* WVP (g∙mm/m^2^∙kPa∙h)	* WS (%)	Moisture (%)	Opacity
1	2.5	35	0.061	1.63	263.6	0.062	3.61	1.01	2.56
2	2.29289	30	0.058	1.19	165.4	0.056	10.36	0.9	2.66
3	2.5	25	0.058	1.11	197.6	0.059	7.85	0.75	2.67
4	3	30	0.078	0.93	152.0	0.077	8.71	1.08	2.17
5	3	30	0.073	0.86	173.9	0.074	10.19	0.76	2.33
6	3	30	0.072	0.66	127.1	0.073	18.94	0.91	2.35
7	3	30	0.073	0.68	92.8	0.071	8.81	0.78	2.34
8	3.70711	30	0.088	0.85	80.8	0.090	12.92	1.01	2.14
9	3	37.0711	0.077	1.02	187.4	0.079	14.84	1.57	2.36
10	3	30	0.073	1.02	151.6	0.074	13.74	0.97	2.32
11	3.5	25	0.084	0.68	177.8	0.083	19.13	1.05	1.95
12	3.5	35	0.088	1.23	149.2	0.091	10.12	1.29	2.14
13	3	22.9289	0.069	1.04	78.8	0.067	8.04	0.58	2.30

* WS is Water Solubility; WVP is Water Vapor Permeability.

**Table 2:** Analysis of variance using ANOVA.

Response	Model	Regression	F-Model/Lack of Fit	*p*-Value Model/Lack of Fit	Model/Lack of Fit
Thickness (mm)	Linear	0.9655	140.14/0.5646	<0.0001/0.7470	Significant/not significant
Opacity	Linear	0.8482	27.94/0.0094	<0.0001/0.3288	Significant/not significant
Moisture (%)	Cubic	0.9074	7.00/0.0112	0.0239/0.9208	Significant/not significant
Water Vapor Permeability (gmm/m^2^∙day∙kPa)	Linear	0.973	180.31/0.5573	<0.0001/0.7513	Significant/not significant

**Table 3:** Equation for responses.

Responses	Equation ^1^
Thickness (mm)	−0.012074 + 0.023857A + 0.000458B
Opacity	+3.62057 − 0.468688A + 0.003820B
Moisture (%)	+0.9000 + 0.03889A + 0.35001B − 0.0050AB + 0.0300A^2^ + 0.0900B^2^ − 0.2250A^2^B + 0.1061AB^2^ + 0.0000A^3^ + 0.0000B^3^
Water Vapor Permeability (g∙mm/m^2^∙kPa∙h)	−0.022135 + 0.025054A + 0.000686B

^1^A: starch concentration. B: glycerol concentration.

## Data Availability

Not applicable.

## Supplementary Materials

The following supporting information can be downloaded at: https://www.mdpi.com/article/10.3390/polym15020338/s1, Table S1: Analysis of variance using Anova; Figure S1: Water contact angle of taro peel starch film.
