# Supplementary material for: Characterization of Biodegradable Films Made from Taro Peel (Colocasia esculenta) Starch
Source: Polymers (Basel). 2023 Jan 9;15(2):338. doi: 10.3390/polym15020338 (PMC9862323; doi:10.3390/polym15020338)
Supplement: Supplementary file 1 [file polymers-15-00338-s001.zip › polymers-2138787-supplementary.pdf]

Supplementary File

Characterization of Biodegradable Films made from Taro Peel (*Colocasia esculenta*) Starch

Rusta Bidari[1], Annur Ahadi Abdillah[2,3], Rogelio Alfredo Bonilla Ponce[2], Albert Linton Charles[2*]

[1] International Master Degree Program in Food Science, National Pingtung University of Science and Technology,1 Shuefu Road, Neipu, Pingtung, Taiwan 91201

[2] Department of Tropical Agriculture and International Cooperation, National Pingtung University of Science and Technology,1 Shuefu Road, Neipu, Pingtung, Taiwan 91201

[3] Department of Marine, Faculty of Fisheries and Marine, Universitas Airlangga, Campus C UNAIR, Mulyorejo, Surabaya, Indonesia 60115

* Correspondence: alcharles@mail.npust.edu.tw

Table S1. Analysis of variance using Anova

Response	Model	Regression	F-model/lack of fit	P-value model/lack of fit	Model/lack of fit
Water Solubility (%)	Linear	0.2467	1.64/0.9220	0.2425/0.5585	Not significant/not significant
Tensile Strength (MPa)	Linear	0.4042	3.39/2.83	0.0751/0.1664	Not significant/not significant
Elongation at Break (%)	Linear	0.3986	3.31/2.65	0.0787 / 0.1823	Not significant/not significant

Figure S1:

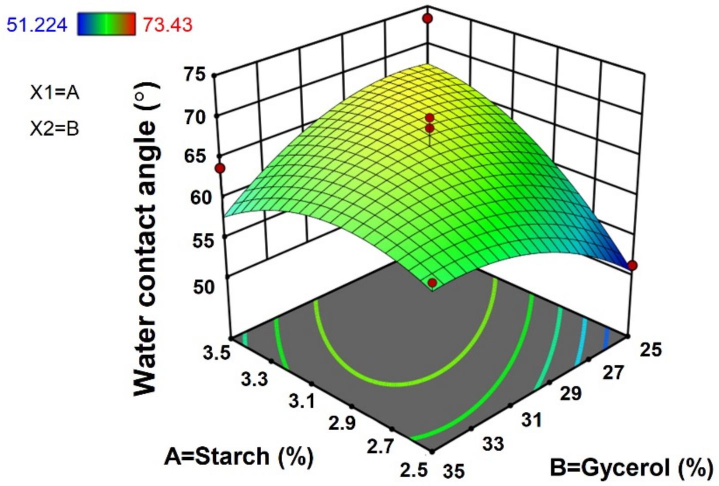

Figure S1. Water contact angle of taro peel starch film.

The water contact angle (WCA) is one of the important parameters to characterize the surface wettability of films as food packaging. The contact angle measured for the films determines whether the nature of the material generated is hydrophobic or hydrophilic (Shanmathy et al., 2021) [8].The biodegradable films with contact angle ϴ<90° possess hydrophilic surface (Wan et al., 2023) [28]. The TPS films had water contact angle with range of (51.2° – 73.4°) respectively, which shows the films were hydrophilic in nature. Therefore, the increase in amount of the starch increased the water vapor permeability of the film decreasing the water contact angle.
